# Thyroid Cancer after Exposure to Radioiodine in Childhood and Adolescence: ^131^I-Related Risk and the Role of Selected Host and Environmental Factors

**DOI:** 10.3390/cancers11101481

**Published:** 2019-10-02

**Authors:** Ljubica Zupunski, Evgenia Ostroumova, Vladimir Drozdovitch, Ilya Veyalkin, Viktor Ivanov, Shunichi Yamashita, Elisabeth Cardis, Ausrele Kesminiene

**Affiliations:** 1 Section of Environment and Radiation, International Agency for Research on Cancer, WHO, 69372 Lyon, France; OstroumovaE@iarc.fr (E.O.); kesminienea@visitors.iarc.fr (A.K.); 2 Division of Cancer Epidemiology and Genetics, National Cancer Institute, NIH, U.S. DHHS, Bethesda, MD 20892, USA; drozdovv@mail.nih.gov; 3 The Republican Research Centre for Radiation Medicine and Human Ecology, 246040 Gomel, Republic of Belarus; veyalkin@mail.ru; 4 National Medical Research Radiological Centre of the Ministry of Health of the Russian Federation, Obninsk, 249036 Kaluga Region, Russia; ivk@obninsk.com; 5 Fukushima Medical University, Fukushima 960-1295, Japan; shun@nagasaki-u.ac.jp; 6 ISGlobal-Barcelona Institute for Global Health, 08003 Barcelona, Spain; elisabeth.cardis@isglobal.org

**Keywords:** Chernobyl nuclear accident, Iodine-131, absorbed thyroid dose, childhood exposure, thyroid cancer, iodine deficiency

## Abstract

In this study, we expanded on a previously published population-based case-control study on subjects exposed to iodine-131 (^131^I) from Chernobyl fallout at age ≤18 years using improved individual ^131^I absorbed thyroid doses. We further studied the impact of iodine deficiency and other selected host risk factors on ^131^I-related thyroid cancer risk after childhood exposure. We included 298 thyroid cancer cases and 1934 matched controls from the most contaminated regions of Belarus and the Russian Federation. We performed statistical analysis using conditional logistic regression models. We found a statistically significant linear quadratic dose-effect association between thyroid cancer and ^131^I thyroid dose in the range up to 5 grays (Gy). Self-reported personal history of benign nodules, any thyroid disease except thyroid cancer, family history of thyroid cancer, increased body mass index, and deficient stable iodine status at the time of the accident were statistically significant risk factors (*p* < 0.05 for each factor) for thyroid cancer after adjustment for thyroid ^131^I dose effect. Subjects who received stable iodine supplementation in the years after the accident had a significantly lower ^131^I-related risk of thyroid cancer. Our findings are important for thyroid cancer prevention, and for further improvement of medical surveillance in the affected populations.

## 1. Introduction

Ionizing radiation is one of the established risk factors for thyroid cancer, especially after exposure during childhood. Evidence of increased radiosensitivity of the thyroid in childhood and adolescence is available not only for external gamma- and x-ray radiation but also for internally incorporated radioiodines [1,2,3,4,5,6]. Excessive risk of thyroid cancer remains elevated 50 or more years after exposure in the atomic bomb survivors who were below 20 years old at the time of bombardments, and 30 years after the Chernobyl radioactive fallout in the Ukrainian-American cohort of people who were below 18 years at the time of the accident [4,6].

The Chernobyl nuclear power station accident resulted in a widespread release of substantial amounts of radionuclides into the atmosphere leading to exposure, predominantly to radioiodines, of large numbers of children and adolescents residing in the most contaminated areas of Belarus, Ukraine, and the Russian Federation (RF). Post-Chernobyl epidemiological studies had reported an increased risk of thyroid cancer, benign thyroid tumours, and thyroid dysfunction, mainly due to internal exposure to iodine-131 (^131^I), although interaction between ^131^I exposure and other potential non-radiation risk factors remained unclear [1,2,3,6,7,8,9].

A population-based case–control study of thyroid cancer was carried out by the International Agency for Research on Cancer (IARC, WHO), in collaboration with the Sasakawa Memorial Health Foundation, in the most contaminated areas of Belarus and Russia [1]. The study aimed to evaluate an association between exposure to ^131^I and risk of thyroid cancer, and the effect of stable iodine supplementation for goiter prophylaxis on the radiation-related thyroid cancer risk among those exposed in childhood and adolescence. This study is, to our knowledge, the only analytical study that used a weighted soil iodine level in the residence of study participants as an environmental indicator of long-term stable iodine intake status. Results of the first analyses, focused on subjects who were less than 15 years old at the time of the Chernobyl accident, were published by Cardis et al. [1]. Recently, some improvements were implemented in the ^131^I dose reconstruction approach by incorporating revised age-specific thyroid mass values for the Belarusian children. 

Since the contribution of radioisotopes other than ^131^I to the thyroid dose was very small and there were no changes in the methodology, we report only on the updated ^131^I thyroid dose–effect analysis using improved ^131^I thyroid dosimetry and on association between non-radiation factors, such as soil iodine deficiency, iodine supplementation, self-reported personal and family history of thyroid diseases, body mass index (BMI), and thyroid cancer risk, and also on their interaction with ^131^I-related thyroid cancer risk. In the current analyses, we also included study subjects who were 15–18 years old at the time of the accident.

## 2. Results

Selected characteristics of the study participants are presented in Table 1. In total, 298 thyroid cancer cases and 1934 controls were included in the study from two regions of Belarus (Gomel, Mogilev) and four regions of the RF (Bryansk, Kaluga, Orel, Tula). One case, confirmed as follicular adenoma by the study international pathologist panel, was excluded from the data analysis, together with the respective controls. Approximately 77% of all cases (228 out of 298) were from Belarus, with 192 out of 228 cases from Gomel region (84%). Approximately 63% of thyroid cancers were in women, and 58% of the cases were less than 5 years old at the time of the accident.

**Figure 1:**
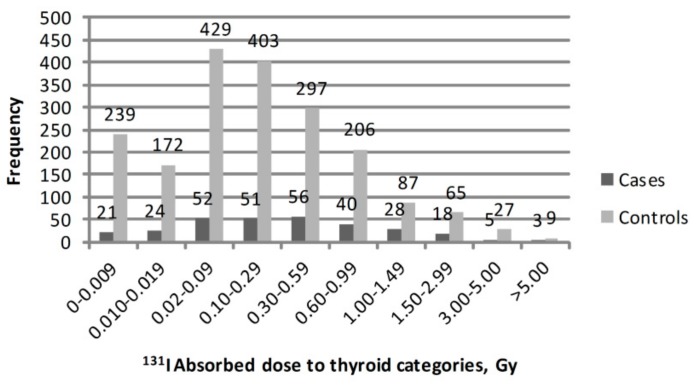
Frequency distribution of study cases and controls by categories of ^131^I absorbed thyroid dose.

^131^I thyroid doses (arithmetic means of 1000 individual stochastic doses) in the study subjects had a log-normal distribution, with 937 subjects having a ^131^I dose <0.10 Gy. The mean (median) value of ^131^I thyroid dose was 0.54 (0.29) Gy for study subjects in Belarus and 0.10 (0.02) Gy in the RF. Mean and median ^131^I thyroid dose estimates by case or control status for six study regions are shown in Table 2. The highest mean ^131^I thyroid doses of 0.77 Gy and 0.58 Gy in cases and controls, respectively, were in Gomel region. Kaluga and Orel regions had the lowest mean ^131^I thyroid dose, which was about 0.03 Gy for both cases and controls. The frequency distribution of study cases and controls by categories of ^131^I absorbed thyroid dose is presented in Figure 1.

Information on thyroid cancer diagnosis circumstances was available for 274 cases. In total, 140 out of 274 cases (51%) were diagnosed at screening, 72 (26%) were diagnosed at doctor’s consultations because of having thyroid-gland-related symptoms, and 62 (23%) were diagnosed at doctor’s consultations after referral for care due to non-thyroid related problems. Information on tumor size was systematically available only for 227 thyroid cancers from Belarus, among which 72 (32%) had tumor size less than 10 mm. Mean (median) tumor size was 14.5 (12) mm, ranging from 2 to 60 mm. Information on cancer staging based on TNM classification of malignant tumors describing tumor size, involvement of lymph nodes and presence of metastasis [10] was available for 227 thyroid cancers from Belarus, among which 151 cases (66.5%) had regional lymph node metastasis, including 5 (3.3%) cases with distant metastasis. 

### 2.1. Association Between ^131^I Thyroid Dose and Thyroid Cancer Risk

Twelve study subjects (3 cases and 9 controls) had estimated doses of above 5 Gy and were excluded from the analysis because we believed that their doses might be overestimated based on the comparison between model-based and measurement-based doses. Subsequently, we also had to exclude 15 control subjects who were matched to those three cases with doses higher than 5 Gy. Additionally, one case and three controls were excluded from the risk analysis because they were in the Ukraine on the April 26, 1986. The 6 respective controls to this case were excluded as well. Overall, 2195 subjects (294 cases and 1901 controls) were included in the thyroid cancer risk analysis. 

Adjustment for self-reported personal history of benign nodules significantly improved the model goodness of fit (*p* < 0.05), so we retained this parameter in the model as a potential confounder of association between ^131^I thyroid dose and thyroid cancer.

**Figure 2:**
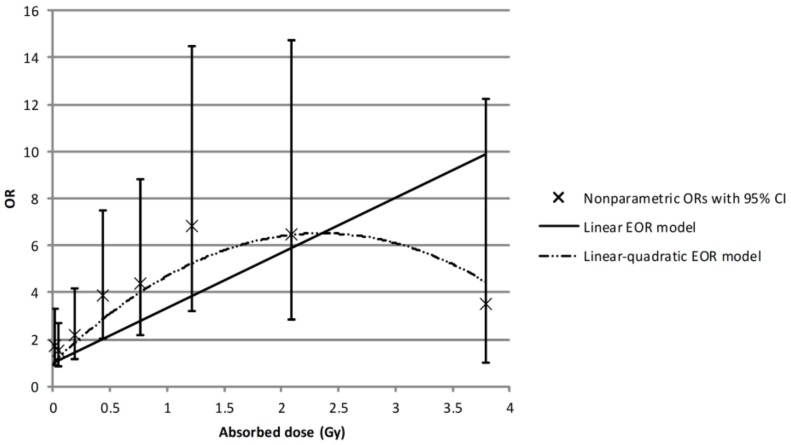
Association between ^131^I thyroid dose and thyroid cancer, risk adjusted for self-reported personal history of benign nodules in the study subjects with ^131^I thyroid absorbed doses <5 Gy. Note: OR = odds ratio; EOR = excess odds ratio

Dose–effect associations between ^131^I thyroid dose and thyroid cancer based on parametric and non-parametric risk models are shown in Figure 2. We found a statistically significant linear dose–effect association with odd ratios (OR) at 1 Gy of 3.34 (95% CI: 2.04; 5.93), based on parameter estimates of β = 2.34 with *p* for linear trend = 0.003. However, similar to the previously reported results, we observed a departure from linearity at a higher dose range from 2 to 5 Gy, where the linear quadratic model described the dose–response better than a simple linear model (*p* test for non-linearity < 0.001), with somewhat higher OR at 1 Gy of 4.71 (95% CI: 2.23; 7.19) based on parameter estimates of β = 4.70 and ϒ = −1.00. At the ^131^I thyroid dose range up to 2 Gy, there was no evidence of non-linearity in the dose response (*p* = 0.49), and OR at 1 Gy was 5.12 (95% CI: 2.98; 9.41), based on parameter estimates of β = 4.12. Thyroid cancer OR estimates by ^131^I thyroid dose categories based on non-parametric dose response analysis are presented in Table 3. ORs were statistically significantly elevated for ^131^I thyroid dose categories above 0.1 Gy, as compared with the reference category (*p* < 0.05 in each case category above 0.1 Gy).

We performed a sensitivity analysis of thyroid cancer risk using the same excess odds ratio (EOR) linear quadratic model and dose range <5 Gy, but excluding 14 thyroid cancer cases having non-papillary subtypes (13 follicular and 1 medullar). We estimated an OR at 1 Gy of 4.58 (95% CI: 2.14; 7.03), showing almost no effect of exclusion of non-papillary thyroid cancers on thyroid cancer radiation risk estimate.

We also performed sensitivity analysis of risk estimates by tumor diameter (<10 mm vs. ≥10 mm) for cases from Belarus where information on tumour size was available (total of 223 cases in the dose range up to 5 Gy). Fitting the EOR model with linear quadratic dose response in the dose range up to 5 Gy, risk estimates were very similar for two groups: the OR at 1 Gy was 5.19 (95% CI: 1.67; 8.71) when cases with tumor size ≥10 mm were included (151 cases), compared to 5.17 (95% CI: 0.13; 10.48) when cases with tumor size smaller than 10 mm were included (72 cases). We did not see statistically significant association between thyroid dose and tumor diameter (*p* = 0.68 based on Spearman’s rank-order correlation test).

### 2.2. Association Between Selected Non-Radiation Risk Factors and Thyroid Cancer

We investigated associations between thyroid cancer and several independent non-radiation risk factors while adjusting for thyroid dose effect. Selected risk factors included self-reported personal and family history of thyroid disease, BMI, stable iodine intake status described by the average level of soil iodine in the settlement at the time of the accident, and iodine supplementation received in months and years following the accident (Table 4). We studied the role of independent risk factors in the subjects with ^131^I thyroid doses up to 2 Gy where there was no statistical evidence of non-linearity in the dose response. We found statistically significant associations between: thyroid cancer and self-reported personal history of benign nodules, with an OR of 14.26 (95% CI: 4.50; 45.18); history of any thyroid disease (except thyroid cancer), with an OR of 1.98 (95% CI: 1.30; 3.00); and family history of thyroid cancer, with an OR of 3.37 (95% CI: 1.38; 8.24). The overweight BMI category was also statistically significantly associated with thyroid cancer, with an OR of 1.87 (95% CI: 1.24; 2.82) (Table 4). 

Both iodine supplementation received regularly at school in the months and years following the accident and stable iodine intake status estimated by the average level of soil iodine in the settlement at the time of the accident showed a statistically significant association with thyroid cancer risk (Table 4). Iodine supplementation had an inverse association with thyroid cancer risk, with an OR of 0.41 (95% CI: 0.25; 0.66) among subjects who consumed antistrumin compared to subjects who did not. The length of antistrumin consumption was less than one year for about 74% of cases and 68% of controls who reported on intake. The thyroid cancer OR estimate in subjects with deficient iodine intake status was 63% higher as compared to those with sufficient iodine intake (*p* = 0.002).

We observed elevated but non-statistically significant thyroid cancer ORs in subjects with self-reported personal history of goiter, family history of any endocrine disease, thyroid nodule, or goiter as compared to the subjects who did not report on personal or family history of these diseases (Table 4).

### 2.3. Effect Modification of the Radiation Dose Response

We tested for possible interaction between ^131^I thyroid dose and a few selected non-radiation risk factors (Table 5). There was no statistically significant variation of ^131^I-related thyroid cancer risk between men and women and by age at the accident (*p* for heterogeneity = 0.78 and 0.15, respectively). 

Iodine supplementation in the years after the accident had a statistically significant modifying effect on ^131^I-related risk of thyroid cancer (*p* = 0.05 for heterogeneity). Subjects who received iodine supplementation in the years after the accident had a significantly lower ^131^I-related risk of thyroid cancer with OR at 1 Gy of 0.65 (95% CI: 0.23; 1.81), while subjects with no iodine supplementation had an OR at 1 Gy of 3.62 (95% CI: 2.43; 5.40). 

Non-statistically significantly increased OR for radiation-related risk of thyroid cancer was also observed among subjects with self-reported history of thyroid diseases other than thyroid cancer as compared to the subjects with no thyroid disease history (*p* for heterogeneity = 0.16); in subjects with self-reported history of benign nodules as compared to subjects reported to be nodule-free; in subjects with family history of thyroid cancer as compared to those without family history of thyroid cancer; in overweight subjects as compared to those with normal BMI; and in subjects with deficient iodine intake status at the time of the accident as compared to those with sufficient iodine intake (*p* for heterogeneity >0.5 in each case) (Table 5).

## 3. Discussion

We report a significant positive association between ^131^I thyroid dose and thyroid cancer risk after exposure at ages below 18 years at the time of the Chernobyl fallout using improved thyroid dosimetry and an expanded dataset, following up on the earlier case–control study [1]. ^131^I thyroid dose estimates were revised based on more precise age-specific thyroid mass values in Belarusian and Russian children, updated reduction factors for different types of milk and dairy products, and reclassified shared and unshared dose errors. This resulted in somewhat lower estimates of ^131^I thyroid dose in the study subjects with ^131^I median dose of 0.29 Gy compared to the previously reported median of 0.36 Gy in Belarus, and of 0.02 Gy as compared to the earlier reported median of 0.04 Gy the RF [1]. 

We evaluated the shape and magnitude of the dose response for ^131^I thyroid doses below 5 Gy and below 2 Gy, and potential modifying effect of some non-radiation risk factors on thyroid cancer ^131^I-related risk. In the thyroid dose range up to 5 Gy, a linear quadratic model described data significantly better than a linear model, while there was no evidence of non-linearity in dose response in analysis restricted to ^131^I doses below 2 Gy. Thyroid cancer OR at 1 Gy was 4.7 (95% CI: 2.2; 7.2) for ^131^I doses up to 5 Gy based on the linear quadratic EOR model. This estimate is comparable to the previously reported OR at 1 Gy of 4.9 (95% CI: 2.2; 7.5), estimated over the entire range of total (^131^I + external exposure) thyroid dose (up to 10.2 Gy), where almost 95% of the total dose was due to ^131^I exposure [1]. In addition, we calculated a similar OR at 1 Gy of 5.1 (95% CI: 3.0; 9.4) for ^131^I doses below 2 Gy based on the linear EOR model, compared to the previously reported OR at 1 Gy of 5.2 (95% CI: 2.2; 8.2) for the same model and dose range [1]. It should be emphasized that ORs in the present study were calculated using the mean of 1000 dose realizations for each subject rather than point estimates in the earlier study [1], and hence our risk estimates are adjusted for errors in doses. However, within this study, we did not consider the dosimetric uncertainties in the confidence intervals of the risk estimates. Use of improved dose estimates that resulted in decrease of ^131^I median dose for 0.07 Gy and 0.02 Gy in Belarus and the RF, respectively, did not have a high impact on the calculated ORs compared to previous analyses. 

Our results are consistent with those reported from thyroid cancer and other thyroid disease studies in the Belarusian-American and Ukrainian-American screening cohorts of subjects who were below the age 18 at the time of the Chernobyl accident and who had direct thyroid activity measurements, with doses mainly from exposure to ^131^I [11]. A significant positive linear association between ^131^I thyroid doses up to 10 Gy and thyroid cancer risk was established in the Ukrainian-American cohort with an EOR/Gy of 5.25 (95% CI: 1.70; 27.5)—that is, a OR of 6.25 at 1 Gy—for prevalent cases found during the first round of screening (from 1998 to 2000), and with an excess relative risk per grey (ERR/Gy) of 1.9 (95% CI: 0.43; 6.34)—that is, a relative risk (RR) at 1 Gy of 2.9—for incident cases diagnosed during second through fourth rounds of screening between 2001 and 2007 in Ukraine [3,12]. Statistically significant ^131^I-related thyroid cancer risk persisted in this cohort, even 30 years after the exposure with EOR/Gy of 1.36 (95% CI: 0.39; 4.15)—that is a OR at 1 Gy of 2.36—for cases diagnosed during the fifth cycle of the screening during 2012–2015 [6]. In the Belarusian-American cohort, a linear dose response was demonstrated for ^131^I doses up to 5 Gy with EOR/ Gy of 2.15 (95% CI: 0.81; 5.47)—that is, an OR of 3.15 at 1 Gy—and a linear-exponential dose response at the dose range up to 32.8 Gy, based on thyroid cancer prevalence cases diagnosed during screening in the period from 1996 through 2004 [2]. In a cross-sectional thyroid ultrasound screening study of 2376 residents around the Semipalatinsk nuclear weapon test site exposed before the age of 21 years, there was a positive association between papillary thyroid cancers and thyroid dose from external and internal radiation sources, although this was not statistically significant and was based on a small number of cases (*n* = 21) [13]. No association was reported between combined benign and malignant thyroid-prevalent tumors and ^131^I thyroid exposure in the Ozyorsk city residents exposed in childhood to atmospheric releases of radioiodines by the Mayak nuclear weapon production, although the results were based on five cases only, which lacked estimates of thyroid doses in the exposed population [14,15].

Our risk estimates are also in agreement with thyroid cancer risks after external irradiation in childhood. In a pooled 12-study analysis of thyroid cancer risk after external environmental and medical exposures in childhood (<20 years at exposure), a relative risk (RR) at 1 Gy of 6.5 (95% CI: 5.1; 8.5) was reported with mean time since exposure of 28.9 years [5]. In that analysis, the dose response was statistically significant and positive for thyroid doses up to 0.1 Gy, with no evidence for non-linearity, though a supralinear relation was seen in the 2–4 Gy dose region, which plateaued between 10–30 Gy and decreased at doses of 30 Gy or more. Among members of the Life Span Study (LSS) cohort of atomic bomb survivors who were exposed to acute external gamma- and neutron-radiation at age 10 years and reached the age of 60 years, an increased thyroid cancer risk was reported with ERR/ Gy of 1.28 (95% CI: 0.59; 2.70), with a linear dose response at thyroid doses up to 2 Gy [4]. 

^131^I-related thyroid cancer risk decreases with attained age and time since exposure, however it remains statistically significant [3,6,12]. In our study, where cases were ascertained between 1992–1998 (mean time since exposure of 9.5 years), our reported risk estimates are fairly comparable with the estimates obtained from the first rounds of screening in Belarusian-American and Ukrainian-American cohorts in 1996–2004 and 1998–2000, respectively [2,12].

We found a significant positive association between thyroid cancer risk and self-reported personal history of benign nodules, of any thyroid disease, and family history of thyroid cancer after adjustment for ^131^I thyroid dose effect. 

Importantly, the indicators of iodine deficiency were statistically significantly associated with the risk of thyroid cancer after adjustment for ^131^I thyroid dose effect. Subjects with deficient iodine intake at the time of the accident had a 63% higher risk of thyroid cancer compared to subjects with sufficient iodine intake; subjects who received iodine supplementation in the years following the accident had an almost 60% lower risk of thyroid cancer compared to those who did not. Other Chernobyl-related studies have reported no evidence of statistically significant thyroid cancer background risk in relation to urinary iodine concentration (used as an indicator of iodine intake, which reflects stable iodine consumption only for a short time before the measurement) [2,3]. In our study, we used average stable iodine content in soil—estimated for villages where the study subjects resided—as a stable iodine intake indicator. It reflects long-term iodine consumption, because in the past, locally produced food was the main source of the daily iodine intake in the study areas. We also found a statistically significant positive association between thyroid cancer risk and increased BMI. A statistically significant association between increased BMI in childhood and increased risk of thyroid cancer in adulthood was reported in a large prospective cohort of children who had weight and height measurements in the period from 7 to 13 years of age [16]. A positive association with BMI before the diagnosis was also observed in the case–control study of thyroid cancer risk in French Polynesia [17]. However, in our study, the measurements were performed 12–16 years after the accident and after the thyroid cancer diagnosis, limiting our ability to discuss whether the observed association is causal or not.

Although thyroid nodules are quite common in clinical practice, about 7–15% of them are expected to be malignant [18,19]. Our study showed a strong association between the self-reported personal history of benign thyroid nodules and thyroid cancer risk, with an OR of 14.29 (95% CI: 4.51; 45.25). However, the estimate was based on a small number of subjects (18 subjects in total, among whom 12 were thyroid cancer cases), and we had no means to validate the self-reported thyroid nodule history. In contrast, in the Ukrainian screening cohort, ultrasound-detected nodules were positively but not statistically significantly associated with increased background risk of thyroid cancer (RR = 2.44, 95% CI: 0.96; 6.19) [3]. 

We did not observe statistically significant variation of the ^131^I-related thyroid cancer risk by personal history of any thyroid disease excluding thyroid cancer (*p* = 0.16), nor by family history of thyroid diseases (*p* > 0.5). Although not statistically significantly, thyroid cancer ^131^I-related risk was much higher in the study subjects with self-reported personal history of benign nodules (*n* = 12) as compared to those who did not report on benign nodule history (*n* = 270), with ORs at 1 Gy of 32.61 and 3.03, respectively (*p* for heterogeneity >0.5). In the Belarusian-American thyroid screening cohort, thyroid cancer radiation-related risk was significantly higher in people with nodular or diffuse goiter in anamnesis or detected at screening compared to goiter-free subjects, and in subjects with enlarged thyroid volume compared to those with normal thyroid volume [2]. By contrast, in the Ukrainian-American cohort, the radiation risk did not vary by presence of diffuse goiter, levels of serum thyroglobulin, or urinary iodine [3]. Also, family history of nodular goiter was positively associated with thyroid cancer risk in Belarus (OR = 3.54, *p* < 0.001) [2].

Iodine deficiency has an impact on the dose–effect relationship by increasing ^131^I thyroid uptake and stimulating thyroid cellular activity [20,21]. Elucidation of the role of iodine deficiency on the radiation-induced thyroid cancer risk is very important, particularly in areas of iodine deficiency. Post-Chernobyl thyroid cancer studies, generally, lack reliable indicators of iodine deficiency of the study population at the time of the accident, and use diffuse goiter, thyroglobulin, or urinary iodine levels as an indicator of long-term deficiency, with the latter two reflecting only the most recent dietary intake [2,3]. Similar to earlier published study [1], we showed that ^131^I-related thyroid cancer risk is somewhat higher in subjects with deficient soil iodine levels as compared with subjects with sufficient soil iodine levels, although in our study, the difference was not statistically significant.

Iodine supplementation affects iodine deficiency status. Since the areas contaminated after Chernobyl were known for being iodine-deficient, regional endocrinological dispensaries, particularly in Belarus, had stocks of potassium iodide (called antistrumin), which was used for goiter prophylaxis in the former Soviet Union. Antistrumin was mainly administered to evacuated children in Belarus, and only a few subjects are reported to have taken it in Russia. The distribution sometimes continued months and even years after the accident [1,22]. Our data showed that subjects who reported regular iodine supplementation in schools and summer camps after the Chernobyl accident had lower risk of spontaneous thyroid cancer compared to the subjects who did not. More importantly, our results suggested that the iodine supplementation received in the years following the accident also diminished the ^131^I-related thyroid cancer risk. As the study population was not systematically provided with stable iodine immediately after the accident, it is plausible that longer-term stable iodine supplementation in the deficient areas could have had a positive effect on the process of thyroid growth in children, resulting in a lower thyroid cancer incidence among them [1]. This has important implications for radiation protection policy, particularly in areas of iodine deficiency in cases of nuclear accidents.

Our study has some limitations, including possible recall bias as a consequence of conducting interviews with the study subjects or their mothers 12–16 years after the accident. Recall bias may have led to under- or overestimation of reported milk and dairy product consumption, resulting in under- or overestimation of individual thyroid doses. It is difficult to evaluate whether such an error would be differential between cases and controls. Even if it is, however, because the thyroid dose reconstruction model depends on many other factors besides level of consumption, it is not clear what effect this would have on the dose–response relationship. Uncertainty in reported consumption is, however, a source of classical error in dose estimations, and would tend to bias risk estimates towards the null in continuous analyses. Another study limitation includes self-reported information on thyroid diseases when the cases could have a better recall than the controls, or when the case may have been under increased surveillance for thyroid cancer. Our assessment of ^131^I-related thyroid cancer risk variation by categories of potential effect modifiers was sometimes limited by relatively small numbers in some categories, preventing detection of interactions. 

Comparison between the model-based individual thyroid doses and the doses based on individual direct thyroid measurements for 64 study subjects with available individual measurements showed a rather wide range of ratios between the two sets of doses [23]. For 70.3% individuals, the correspondence between the two doses was within a factor of 3. The mean ± standard deviation of ratios of thyroid dose based on the model to the dose based on direct thyroid measurements was found to be 1.2 (±1.3; median of 0.8). There was a moderate positive correlation between model-based individual thyroid doses and the doses based on individual direct thyroid measurements (Spearman’s correlation coefficient = 0.50, *p* < 0.001). The observed difference between model- and measurement-based thyroid doses could be explained by relatively large uncertainties in the doses calculated using the “semi-empirical” model and uncertainties associated with recalling the information on relocation history and individual diet of the study subjects collected more than 10 years after the Chernobyl accident. To account for the uncertainties associated with parameters of the semi-empirical model, thyroid mass-values, ^131^I deposition densities, and imprecise responses to questions administered during the personal interview, a set of 1000 individual stochastic doses was generated using MC simulation for each study individual [23]. We did not further address the dose uncertainty in dose–effect analysis. 

## 4. Materials and Methods 

### 4.1. Study Design

A population-based case–control study was conducted to evaluate thyroid cancer risk in people who were below 18 years old at the time of the Chernobyl accident and resided in the territories heavily contaminated after the radioactive fallout in Belarus and the RF. The present analysis included 298 thyroid cancer cases and 1934 matched control subjects—residents of Gomel and Mogilev regions of Belarus, and of Bryansk, Kaluga, Orel, and Tula regions of the RF. 

The detailed study methodology, including case ascertainment and controls selection, is reported elsewhere [1]. In brief, thyroid cancer patients had histologically verified thyroid carcinoma diagnosed between January 1, 1992, and December 31, 1998, and underwent surgery in the RF or Belarus. Cases were identified from multiple sources: the population-based cancer registry; the Russian national medical and dosimetry registry; Republican Scientific and Practical Center for Thyroid Tumors, Belarus; and the oncological dispensaries and surgical departments in the study regions of both countries. Histological slides from the study cases were reviewed by the international panel of pathologists, who confirmed that the majority of tumors were papillary carcinomas; 284 thyroid cancers were confirmed as papillary carcinomas, 13 as follicular carcinomas, one as medullar carcinoma and one as follicular adenoma. At least 5 control subjects were matched to each case by age, sex, and administrative region of residence. Controls were drawn randomly from birth registry records covering the population at the administrative regional level. In Kaluga and Orel regions (in the RF), computerized medical insurance system records were used. 

A personal interview was performed with study subjects and with the study subject’s mother (if the subject was less than 12 years old at the time of the accident) by trained interviewers using a detailed questionnaire. At the interview, information was collected on places and dates of residence at the time of the accident and following the accident, dietary habits and lifestyle factors, iodine blocking received immediately after the accident and stable iodine supplementation in the months and years following the accident, and history of thyroid diseases of study subjects and their family. Stable iodine supplementation was administrated in the form of antistrumin (potassium iodide) tablets given to children at schools and summer camps and used for goiter prophylaxis in the former Soviet Union. 

Each study participant or their legal guardian provided written informed consent to participate in the study. The study was approved by the International Agency for Research on Cancer (IARC) Ethics Review Committee (ethic code: 98-007), the Belarus Coordinating Council for Studies of the Medical Consequences of the Chernobyl Accident, and the Ethical Committee of A. Tsyb Medical Radiological Research Centre, a branch of the National Medical Radiological Research Centre of the Ministry of Health of the Russian Federation, Obninsk. 

### 4.2. Thyroid Dose Assessment

Individual absorbed dose to the thyroid from ^131^I was reconstructed for each study subject for the following pathways of exposure: (*i*) inhalation of contaminated air; (*ii*) consumption of contaminated milk (typically the most important pathway), milk products, and leafy vegetables from April 26 until June 20, 1986. The period was established based on ^131^I decay pattern, with a half-life of about 8 days.

The semi-empirical model used to estimate individual thyroid dose due to ^131^I intake is based on the relationship between environmental contamination (^131^I deposition) and thyroid dose, estimated from individual direct measurements of thyroid radioactivity carried out in territories with different contamination levels and in the population with all age groups. The model allows for estimation of the so-called “standard thyroid dose” for an adult in a given settlement under the assumptions of standard diet, the absence of any countermeasures, and the permanent residence of the person in the settlement. The model was further modified to estimate individual thyroid doses using study questionnaire information on individual behaviors, including residential history, dietary habits (origin and consumption of milk, milk products, leafy vegetables), and potassium iodine administration to block thyroidal uptake of ^131^I shortly after the accident [24]. 

Recently, we further improved the precision of thyroid dose assessment in the study by using: (*i*) region-, sex-, and age-specific thyroid mass values for Belarusian [25] and Russian [26] children; (*ii*) characterization of distribution of settlement-specific ^137^Cs deposition densities and ratios of ^131^I to ^137^Cs activities in deposition obtained from radiation measurements data that had been collected and systemized [27]; and (*iii*) proper separation of sources of shared and unshared errors in dosimetry models [28]. 

We used a Monte Carlo (MC) simulation procedure to calculate 1000 individual stochastic thyroid doses for each subject, accounting for shared and unshared sources of errors. A detailed description of the dosimetry model and methods used to improve thyroid dose assessment in the study can be found elsewhere [23].

To validate the dosimetry model used to estimate the thyroid doses in this study, we compared a sample of ^131^I thyroid doses estimated based on the dosimetry model, with the doses estimated based on direct measurements of ^131^I thyroidal activity in those study subjects who had both model-based and measurement-based thyroid doses (*n* = 64) [23]. 

### 4.3. Study Indicators of Iodine Sufficiency 

We assessed iodine sufficiency level for each study subject based on the information from personal interviews at two time points: at the time of the Chernobyl accident and at the time of diagnosis of the case within each match set. The level of stable iodine content in soil in the settlement where the subject resided at the time of the accident and the weighted mean of the soil iodine content in different settlements where this subject lived in the time period after the accident until the year of thyroid cancer diagnosis of the case were assessed based on the soil characteristics determined from detailed maps, and used as indicators of stable iodine intake. The method is described in detail in Korobova et al. 2010 [29]. In addition, different weights were applied for each settlement based on their rural or urban status, because of the fact that urban and rural settlements had different food supply characteristics (industrial or domestic production). For rural settlements, it was assumed that the population consumed mostly foodstuffs produced within the same area, so the level of soil content of iodine reflected their stable iodine intake status. For large cities, it was assumed that the population was iodine-sufficient because people mostly consumed imported foodstuffs from regions that were considered as iodine-sufficient. For towns, it was assumed that locally produced foodstuffs contributed with 50% to the diet, while 50% were imported. We considered stable iodine intake status as deficient when iodine in the soil in a settlement was <2.5 mg/m^3^ and sufficient when the iodine level was ≥2.5 mg/m^3^. The cut-off point was taken to be the lowest tertile of the stable iodine level in soil. 

### 4.4. Statistical Analysis

We analyzed the data using conditional logistic regression models. In the continuous analysis, the odds ratio (OR) was expressed as:

(1) $$OR\left(d\right)=e^{\alpha x}\left(1+\beta d+\gamma d^{2}\right)$$

where *x* represents a vector of covariates considered as potential confounders, *d* represents the thyroid dose, and *α*, *β*, and *ϒ* are unknown parameters. The sum of the βd linear dose response term and $\gamma d^{2}$ quadratic dose response term represents the excess odds ratio (EOR). 

We used the mean dose of 1000 dose realizations for each subject in the dose–response analysis. We fitted a linear dose response model using thyroid dose as a continuous variable. We also tested a departure of dose response from linearity by adding a quadratic term $\left(\gamma d^{2}\right)$ to Equation (1). We used the likelihood ratio test (LRT) to compare goodness of fit of the linear quadratic versus the simple linear dose–response model. 

We also fit dose as a categorical variable using a non-parametric log-linear dose–response model. Dose categories were chosen to span the width of the skewed log-normal distribution of doses with the following dose category cut-off points: 0/0.010/0.02/0.10/0.30/0.60/1.00/1.50/3.00 + Gy. Calculated ORs and 95% confidence intervals (CI) represent the risk of thyroid cancer in a given dose interval compared to the reference category.

We limited our dose response analysis to the study subjects with ^131^I thyroid dose below 5 Gy, because based on the comparison between model-based and measurement-based dose estimates, we assumed that their dose could be overestimated. 

We assessed effects of several potential confounding factors on the association between thyroid dose and thyroid cancer risk. Potential confounding factors included self-reported personal history of any thyroid disease except thyroid cancer, self-reported personal history of benign nodules, family history of thyroid cancer, BMI, stable iodine supplementation in years after the accident, and stable iodine status at the time of the accident. The factor was considered as a potential confounder if EOR/ Gy changed by more than 10%, or if adding the factor to the model significantly improved the model fit (*p*-value of < 0.05 based on LRT to compare goodness of fit of the models with or without potential confounder). 

To test for associations between independent non-radiation factors and thyroid cancer risk, and also to assess for possible effect modification of radiation-induced risk of thyroid cancer, we used a log-linear OR model to ensure stable convergence of the model. The log-linear OR model was expressed as:

(2) $$OR\left(d\right)=e^{\beta d+\gamma z+\delta \left(d\times z\right)}$$

where *d* represents thyroid dose; *z* represents the independent non-radiation risk factor and potential dose response modifier; *d×z* represents the interaction term between the dose and potential effect modifier; and *β*, *ϒ*, and *δ* represent unknown parameters. Non-radiation risk factors included sex, age at the time of the accident, self-reported personal history of any thyroid disease except thyroid cancer, self-reported history of benign nodule or goiter, family history of thyroid or other endocrine disease, family history of benign nodules, family history of goiter, iodine supplementation in years after the accident, stable iodine intake status, and BMI. We calculated BMIs based on study subjects’ weight and height measured during clinical examination performed around the time of personal interview, which was in the years from 1998 through 2002, by dividing body weight (in kilograms) by height (in meters) squared. For data analysis, we placed study subjects’ BMI estimates into two categories (“normal” versus “overweight”) using sex- and age-specific reference cut-off values, defined by the World Health Organization (WHO) growth curves for school-aged children and adolescents (5–19 years old) [30]. The number of underweight and obese subjects in the study was very small, so we combined underweight with the normal category, and obese with the overweight category. 

We used the PECAN module of the EPICURE version 1.81 statistical software for matched case-control data to fit the models [31]. All statistical tests were two-sided, with p-values of ≤0.05 considered as statistically significant. The 95% CIs were calculated using the maximum likelihood method, except for the linear quadratic EOR model and the log-linear model, where Wald bounds were calculated. 

## 5. Conclusions

The analysis presented here, using improved thyroid dose estimates, confirms the previous findings of this and other studies concerning the effect of ^131^I on thyroid cancer risk after childhood radiation exposure (≤18 years of age at exposure). As before, our more detailed analysis suggested that iodine supplementation in iodine-deficient areas has the potential to decrease ^131^I-related thyroid cancer risk in people exposed in childhood. The results are important for improvement of radiation protection policies in cases of future nuclear accidents and of medical surveillance of the affected population. Further research is needed to gain more insights into the underlying biological mechanisms of radiation-induced thyroid cancer development, including the role of benign nodules, as well as iodine deficiency.

## Figures and Tables

**Table 1:** Descriptive characteristics of study cases and controls.

Characteristics	Cases	Controls	Total
Total	298	1934	2232
Sex			
Male	109	700	809
Female	189	1234	1423
Country/Region			
Belarus			
Gomel	192	1239	1431
Mogilev	36	230	266
Russian Federation			
Bryansk	11	74	85
Kaluga	10	60	70
Orel	27	187	214
Tula	22	144	166
Age at exposure, years			
<2	89	699	788
2–4	84	520	604
5–9	67	364	431
10–14	44	247	291
15–18	14	104	118

**Table 2:** Iodine-131 (^131^I) thyroid dose estimates by case or control status and study region, in grays (Gy).

Region	Median Dose for Cases	Median Dose for Controls	Mean Dose for Cases	Mean Dose for Controls
Belarus				
Gomel	0.51	0.31	0.77	0.58
Mogilev	0.11	0.056	0.48	0.14
Russian Federation				
Bryansk	0.35	0.086	0.80	0.40
Kaluga	0.011	0.019	0.031	0.031
Orel	0.014	0.018	0.028	0.031
Tula	0.019	0.021	0.037	0.036
Total	0.31	0.15	0.59	0.41

**Table 3:** Thyroid cancer odds ratios (ORs) and 95% confidence intervals (95% CI) by ^131^I thyroid dose categories.

Dose Category, Gy	Mean Dose, Gy	Number of Cases	OR *	95% CI
0–0.009	0.005	21	1.00	Reference
0.010–0.019	0.014	24	1.71	0.89; 3.29
0.02–0.09	0.05	52	1.51	0.85; 2.70
0.10–0.29	0.19	50	2.21	1.17; 4.19
0.30–0.59	0.43	56	3.88	2.01; 7.50
0.60–0.99	0.77	40	4.38	2.17; 8.83
1.00–1.49	1.22	28	6.83	3.22; 14.48
1.50–2.99	2.10	18	6.49	2.86; 14.75
3.00–5.00	3.77	5	3.53	1.02; 12.22

* Model adjusted for self-reported personal history of benign nodules.

**Table 4:** Thyroid cancer odds ratios (OR) and 95% confidence intervals (95% CI) for selected risk factors for study subjects with ^131^I thyroid doses <2 Gy.

Variable	*N* of Cases (%)	*N* of Controls (%)	OR ^a^ (95% CI)
Personal history of thyroid diseases			
Any thyroid disease except thyroid cancer			
Never had	246 (87.2)	1680 (92.6)	1.00 (Referent)
Ever had	36 (12.8)	134 (7.4)	1.98 (1.30; 3.00)
Benign nodules			
no	270 (95.7)	1808 (99.7)	1.00 (Referent)
yes	12 (4.3)	6 (0.3)	14.26 (4.50; 45.18)
Goiter			
no	246 (92.1)	1680 (93.4)	1.00 (Referent)
yes	21 (7.9)	118 (6.6)	1.29 (0.78; 2.15)
Family history of thyroid diseases			
Thyroid cancer			
no	266 (96.7)	1745 (99.0)	1.00 (Referent)
yes	9 (3.3)	18 (1.0)	3.37 (1.38; 8.24)
Thyroid or other endocrine disease ^b^			
no	200 (74.4)	1322 (77.2)	1.00 (Referent)
yes	69 (25.6)	391 (22.8)	1.22 (0.90; 1.66)
Nodule			
no	248 (94.3)	1639 (96.3)	1.00 (Referent)
yes	15 (5.7)	63 (3.7)	1.51 (0.82; 2.77)
Goiter			
no	228 (87.0)	1536 (90.3)	1.00 (Referent)
yes	34 (13.0)	166 (9.7)	1.33 (0.88; 2.00)
Anthropomorphic factor			
BMI			
Normal	117 (73.6)	1385 (84.4)	1.00 (Referent)
Overweight	42 (26.4)	257 (15.7)	1.87 (1.24; 2.82)
Stabile iodine status			
Iodine supplementation			
no	241 (91.3)	1304 (83.1)	1.00 (Referent)
yes	23 (8.7)	265 (16.9)	0.41 (0.25; 0.66)
Stabile iodine intake status at the time of the accident ^c^			
Deficient	118 (41.8)	548 (29.8)	1.63 (1.20; 2.21)
Sufficient	164 (58.2)	1293 (70.2)	1.00 (Referent)

^a^ Odds ratios calculated using log-linear model in the form $OR\left(d\right)=e^{\beta d+\gamma z}$, where d represents the thyroid dose and z represents independent non-radiation risk factor. Individual absorbed dose to the thyroid from ^131^I was reconstructed for the period from April 26 until June 20, 1986. ^b^ Other endocrine disease includes diabetes, goiter, thyroiditis, and obesity. ^c^Deficient stabile iodine intake status defined as <2.5 mg/m^3^ of iodine in soil; sufficient stabile iodine intake status defined as ≥2.5 mg/m^3^ of iodine in soil. Note: BMI = body mass index.

**Table 5:** Thyroid cancer odds ratios (OR) at 1 Gy and 95% confidence interval (95% CI) by categories of selected potential radiation-effect modifiers for study subjects with ^131^I thyroid doses <2 Gy.

Variable	*N* of Thyroid Cancer Cases	OR at 1 Gy (95% CI) ^a^	*p*-Value for Heterogeneity
Sex			
men	102	3.01 (1.76; 5.14)	0.78
women	180	3.33 (2.06; 5.36)	
Age at the accident, years			
≤1.5	65	2.34 (1.41; 3.92)	
1.6–3.0	61	3.03 (1.74; 5.25)	0.15
3.1–8.0	87	6.22 (3.11; 12.44)	
8.1+	69	3.17 (0.90; 11.17)	
Personal history of thyroid disease except thyroid cancer			
Never had	246	3.44 (2.37; 4.98)	
Ever had	36	4.34 (1.98; 9.49)	0.16
Personal history of benign nodules			
no	270	3.03 (2.12; 4.34)	
yes	12	32.61 (5.77; 184.18)	0.68
Family history of thyroid cancer			
no	266	3.21 (2.23; 4.61)	
yes	9	8.31 (2.11; 32.79)	0.62
BMI			
Normal	117	3.78 (2.19; 6.55)	
Overweight	42	6.80 (2.72; 16.96)	0.92
Iodine supplementation			
no	241	3.62 (2.43; 5.40)	
yes	23	0.65 (0.23; 1.81)	0.05
Stabile iodine intake status at the time of the accident ^b^			
Deficient	118	4.65 (3.01; 7.18)	0.90
Sufficient	164	2.80 (1.66; 4.70)	

^a^ Based on log-linear model in the form: $OR\left(d\right)=e^{\beta d+\gamma z+\delta \left(d\times z\right)}$, where d represents the thyroid dose, z represents independent non-radiation risk factor, d×z represents interaction term between these two variables, and β, ϒ, and δ represent unknown parameters. Individual absorbed dose to the thyroid from ^131^I was reconstructed for the period from April 26 until June 20, 1986. ^b^ Deficient stabile iodine intake status defined as <2.5 mg/m^3^ of iodine in soil; sufficient stabile iodine intake status defined as ≥2.5 mg/m^3^ of iodine in soil.

## References

[1] Cardis E., Kesminiene A., Ivanov V., Malakhova I., Shibata Y., Khrouch V., Drozdovitch V., Maceika E., Zvonova I., Vlassov O. (2005). Risk of Thyroid Cancer after Exposure to 131 I in Childhood. JNCI J. Natl. Cancer Inst..

[2] Zablotska L.B., Ron E., Rozhko A.V., Hatch M., Polyanskaya O.N., Brenner A.V., Lubin J., Romanov G.N., McConnell R.J., O’Kane P. (2011). Thyroid cancer risk in Belarus among children and adolescents exposed to radioiodine after the Chornobyl accident. Br. J. Cancer.

[3] Brenner A.V., Tronko M.D., Hatch M., Bogdanova T.I., Oliynik V.A., Lubin J.H., Zablotska L.B., Tereschenko V.P., McConnell R.J., Zamotaeva G.A. (2011). I-131 dose response for incident thyroid cancers in Ukraine related to the Chornobyl accident. Environ. Health Perspect..

[4] Furukawa K., Preston D., Funamoto S., Yonehara S., Ito M., Tokuoka S., Sugiyama H., Soda M., Ozasa K., Mabuchi K. (2013). Long-Term trend of thyroid cancer risk among Japanese Atomic-Bomb survivors: 60 years after exposure. Int. J. Cancer.

[5] Veiga L.H.S., Holmberg E., Anderson H., Pottern L., Sadetzki S., Adams M.J., Sakata R., Schneider A.B., Inskip P., Bhatti P. (2016). Thyroid Cancer after Childhood Exposure to External Radiation: An Updated Pooled Analysis of 12 Studies. Radiat. Res..

[6] Tronko M., Brenner A.V., Bogdanova T., Shpak V., Oliynyk V., Cahoon E.K., Drozdovitch V., Little M.P., Tereshchenko V., Zamotayeva G. (2017). Thyroid neoplasia risk is increased nearly 30 years after the Chernobyl accident. Int. J. Cancer.

[7] Ostroumova E., Rozhko A., Hatch M., Furukawa K., Polyanskaya O., McConnell R.J., Nadyrov E., Petrenko S., Romanov G., Yauseyenka V. (2013). Measures of thyroid function among Belarusian children and adolescents exposed to iodine-131 from the accident at the Chernobyl nuclear plant. Environ. Health Perspect..

[8] Zablotska L.B., Nadyrov E.A., Polyanskaya O.N., McConnell R.J., O’Kane P., Lubin J., Hatch M., Little M.P., Brenner A.V., Veyalkin I.V. (2015). Risk of thyroid follicular adenoma among children and adolescents in Belarus exposed to iodine-131 after the Chornobyl accident. Am. J. Epidemiol..

[9] Cahoon E.K., Nadyrov E.A., Polyanskaya O.N., Yauseyenka V.V., Veyalkin I.V., Yeudachkova T.I., Maskvicheva T.I., Minenko V.F., Liu W., Drozdovitch V. (2017). Risk of Thyroid Nodules in Residents of Belarus Exposed to Chernobyl Fallout as Children and Adolescents. J. Clin. Endocrinol. Metab..

[10] Greene F.L., Page D.L., Fleming I.D., Fritz A.G., Balch C.M., Haller D.G., Morrow M. (2002). AJCC Cancer Staging Manual.

[11] Stezhko V.A., Buglova E.E., Danilova L.I., Drozd V.M., Krysenko N.A., Lesnikova N.R., Minenko V.F., Ostapenko V.A., Petrenko S.V., Polyanskaya O.N. (2004). A cohort study of thyroid cancer and other thyroid diseases after the Chornobyl accident: Objectives, design and methods. Radiat. Res..

[12] Tronko M.D., Howe G.R., Bogdanova T.I., Bouville A.C., Epstein O.V., Brill A.B., Likhtarev I.A., Fink D.J., Markov V.V., Greenebaum E. (2006). A Cohort Study of Thyroid Cancer and Other Thyroid Diseases After the Chornobyl Accident: Thyroid Cancer in Ukraine Detected During First Screening. JNCI J. Natl. Cancer Inst..

[13] Land C.E., Kwon D., Hoffman F.O., Moroz B., Drozdovitch V., Bouville A., Beck H., Luckyanov N., Weinstock R.M., Simon S.L. (2015). Accounting for shared and unshared dosimetric uncertainties in the dose response for ultrasound-detected thyroid nodules after exposure to radioactive fallout. Radiat. Res..

[14] Khokhryakov V., Drozhko E., Glagolenko Y., Rovny S., Vasilenko E., Suslov A., Anspaugh L., Napier B., Bouville A., Khokhryakov V. (2002). Studies on the Ozyorsk population: Dosimetry. Radiat. Environ. Biophys..

[15] Mushkacheva G., Rabinovich E., Privalov V., Povolotskaya S., Shorokhova V., Sokolova S., Turdakova V., Ryzhova E., Hall P., Schneider A.B. (2006). Thyroid Abnormalities Associated with Protracted Childhood Exposure to 131I from Atmospheric Emissions from the Mayak Weapons Facility in Russia. Radiat. Res..

[16] Kitahara C.M., Gamborg M., Berrington de González A., Sørensen T.I.A., Baker J.L. (2014). Childhood height and body mass index were associated with risk of adult thyroid cancer in a large cohort study. Cancer Res..

[17] Brindel P., Doyon F., Rachédi F., Boissin J.-L., Sebbag J., Shan L., Chungue V., Bost-Bezeaud F., Petitdidier P., Paoaafaite J. (2009). Anthropometric factors in differentiated thyroid cancer in French Polynesia: A Case–Control study. Cancer Causes Control..

[18] Popoveniuc G., Jonklaas J. (2012). Thyroid nodules. Med. Clin. N. Am..

[19] Haugen B.R., Alexander E.K., Bible K.C., Doherty G.M., Mandel S.J., Nikiforov Y.E., Pacini F., Randolph G.W., Sawka A.M., Schlumberger M. (2016). 2015 American Thyroid Association Management Guidelines for Adult Patients with Thyroid Nodules and Differentiated Thyroid Cancer: The American Thyroid Association Guidelines Task Force on Thyroid Nodules and Differentiated Thyroid Cancer. Thyroid..

[20] Hatch M., Polyanskaya O., McConnell R., Gong Z., Drozdovitch V., Rozhko A., Prokopovich A., Petrenko S., Brenner A., Zablotska L. (2011). Urinary Iodine and Goiter Prevalence in Belarus: Experience of the Belarus-American cohort study of thyroid cancer and other thyroid diseases following the Chornobyl nuclear accident. Thyroid.

[21] WHO, UNICEF, ICCIDD (2007). Assessment of Iodine Deficiency Disorders and Monitoring Their Elimination: A Guide for Programme Managers.

[22] United Nations Scientific Committee on the Effects of Atomic Radiation (UNSCEAR) (2000). Sources and Effects of Ionizing Radiation-Vol. II Effects.

[23] Drozdovitch V., Kesminiene A., Moissonnier M., Veyalkin I., Ostroumova E. (2019). Uncertainties in radiation doses for a Case-Control study of thyroid cancer among residents exposed in childhood to 131I from Chernobyl fallout. Health Phys..

[24] Drozdovitch V., Khrouch V., Maceika E., Zvonova I., Vlasov O., Bratilova A., Gavrilin Y., Goulko G., Hoshi M., Kesminiene A. (2010). Reconstruction of radiation doses in a Case-Control study of thyroid cancer following the Chernobyl accident. Health Phys..

[25] Skryabin A.M., Drozdovitch V., Belsky Y., Leshcheva S.V., Mirkhaidarov A.K., Voillequé P., Luckyanov N., Bouville A. (2010). Thyroid mass in children and adolescents living in the most exposed areas to Chernobyl fallout in Belarus. Radiat. Prot. Dosim..

[26] Yamashita S., Shibata Y. (1997). Proceedings of the Fifth Chernobyl Sasakawa Medical Cooperation Symposium, Kyiv, Ukraine, 14–15 October 1996.

[27] Drozdovitch V., Zhukova O., Germenchuk M., Khrutchinsky A., Kukhta T., Luckyanov N., Minenko V., Podgaiskaya M., Savkin M., Vakulovsky S. (2013). Database of meteorological and radiation measurements made in Belarus during the first three months following the Chernobyl accident. J. Environ. Radioact..

[28] Drozdovitch V., Minenko V., Golovanov I., Khrutchinsky A., Kukhta T., Kutsen S., Luckyanov N., Ostroumova E., Trofimik S., Voillequé P. (2015). Thyroid Dose Estimates for a Cohort of Belarusian Children Exposed to (131) I from the Chernobyl Accident: Assessment of Uncertainties. Radiat. Res..

[29] Korobova E., Anoshko Y., Kesminiene A., Kouvyline A., Romanov S., Tenet V., Suonio E., Cardis E. (2010). Evaluation of stable iodine status of the areas affected by the Chernobyl accident in an epidemiological study in Belarus and the Russian Federation. Selenium Iodine Anom. Soils Health.

[30] De Onis M., Onyango A.W., Borghi E., Siyam A., Nishida C., Siekmann J. (2007). Development of a WHO growth reference for School-Aged children and adolescents. Bull. World Health Organ..

[31] Preston D.L., Lubin J.H., Pierce D.A., McConney M.E. (1993). Epicure User’s Guide.

